# Arbuscular Mycorrhizal Fungi Increase Pb Uptake of Colonized and Non-Colonized *Medicago truncatula* Root and Deliver Extra Pb to Colonized Root Segment

**DOI:** 10.3390/microorganisms9061203

**Published:** 2021-06-02

**Authors:** Haoqiang Zhang, Wei Ren, Yaru Zheng, Yanpeng Li, Manzhe Zhu, Ming Tang

**Affiliations:** 1 College of Forestry, Northwest A&F University, Yangling 712100, China; zhanghaoqiang@nwsuaf.edu.cn (H.Z.); renw@nwsuaf.edu.cn (W.R.); zhengyaru@nwafu.edu.cn (Y.Z.); wo982805717@gmail.com (Y.L.); zhumanzhe@gmail.com (M.Z.); 2 State Key Laboratory of Conservation and Utilization of Subtropical Agro-Bioresources, Lingnan Guangdong Laboratory of Modern Agriculture, Guangdong Key Laboratory for Innovative Development and Utilization of Forest Plant Germplasm, College of Forestry and Landscape Architecture, South China Agricultural University, Guangzhou 510642, China

**Keywords:** Pb uptake, translocation, resource allocation, arbuscular mycorrhizal pathway for Pb uptake, transpiration pull

## Abstract

Arbuscular mycorrhizal (AM) fungi establish symbiosis and improve the lead (Pb) tolerance of host plants. The AM plants accumulate more Pb in roots than their non-mycorrhizal counterparts. However, the direct and long-term impact of AM fungi on plant Pb uptake has been rarely reported. In this study, AM fungus (*Rhizophagus irregularis*) colonized and non-colonized roots of *Medicago truncatula* were separated by a split-root system, and their differences in responding to Pb application were compared. The shoot biomass accumulation and transpiration were increased after *R. irregularis* inoculation, whereas the biomass of both colonized and non-colonized roots was decreased. Lead application in the non-colonized root compartment increased the *R. irregularis* colonization rate and up-regulated the relative expressions of *MtPT4* and *MtBCP1* in the colonized root compartments. *Rhizophagus irregularis* inoculation increased Pb uptake in both colonized and non-colonized roots, and *R. irregularis* transferred Pb to the colonized root segment. The Pb transferred through the colonized root segment had low mobility and might be sequestrated and compartmented in the root by *R. irregularis.* The Pb uptake of roots might follow water flow, which is facilitated by MtPIP2. The quantification of Pb transfer via the mycorrhizal pathway and the involvement of MtPIP2 deserve further study.

## 1. Introduction

Heavy metal contamination in soil is a worldwide issue due to rapid urbanization, mining, sewage sludge, application of fertilizers, and other anthropogenic activities [1,2,3]. Lead (Pb) is one of the most common heavy metal pollutants in China [4] and is a non-essential element that poses an immense risk especially for children [5,6]. Phytoremediation is an efficient and non-invasive way to remediate contaminated soils [7,8]. The application of microorganisms in the phytoremediation of Pb has received extensive attention [9,10,11,12].

Arbuscular mycorrhizal (AM) fungi can establish mutualistic symbioses with more than 80% of terrestrial plants in different ecosystems [13,14], including Pb polluted areas [15,16]. With AM fungal colonization, plants usually have higher biomass [17,18], increased antioxidant enzyme activities and photosynthetic rates, and improved Pb tolerance [18,19]. Moreover, the establishment of AM symbiosis leads to the enhancement of host plant photosynthetic rates, transpiration flow, and water uptake [18,20,21,22,23]. The water transport from soil to leaves requires the participation of aquaporins (AQPs), which are a class of membrane intrinsic proteins. Aquaporins mediate water transport across membranes following an osmotic gradient [24] and participate in hydraulic conductance regulation [25,26]. Plant AQPs include plasma membrane intrinsic proteins (PIPs), tonoplast intrinsic proteins (TIPs), NOD26-like intrinsic proteins (NIPs), small basic intrinsic proteins, and uncategorized intrinsic proteins [25]. In AM plants, the uptake of water and nutrients was suggested via two pathways. One pathway (plant root pathway) relies on plant root, and the other pathway (mycorrhizal pathway) relies on the AM fungal hyphae [27,28]. It has been confirmed that nutrient transportation via the mycorrhizal pathway to plants requires the participation of aquaporin [29].

**Figure 1:**
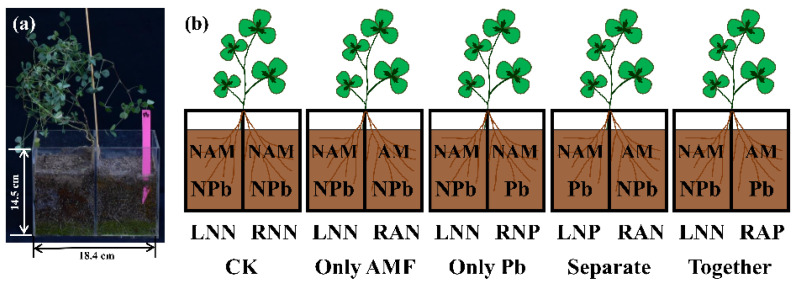
(**a**) Split root system used for the study of the influence of *R. irregularis* on Pb uptake by *M. truncatula*. (**b**) The 2 compartments of the root systems inoculated with/without *R. irregularis*, and 2 compartments with/without Pb applied after 4 weeks of mycorrhizal colonization. NAM = non-mycorrhizal treatment; AM = arbuscular mycorrhizal fungi inoculation; Pb = Pb treatment; NPb = non-Pb treatment. CK = control treatment. Only Pb = Pb was added to only 1 compartment; Only AMF = AM fungi was added to only 1 compartment; Separate = Pb and AM fungi were added into the split-root system in the 2 compartments separately; Together = Pb and AM fungi were added into the split-root system in the same compartment together. LNN = no AM fungi or Pb was added to the left compartment; RNN = no AM fungi or Pb was added to the right compartment; RAN = AM fungi was added to the right compartment without Pb; RNP = Pb was added to the right compartment without AM fungi; LNP = Pb was added to the left compartment without AM fungi; RAP = AM fungi and Pb were added to the right compartment.

In previous studies, the AM plants accumulated more Pb in roots and less Pb in shoots than their non-mycorrhizal counterparts [18,30,31]. However, the direct and long-distance impact of AM fungi on plant Pb uptake has not been reported. *Medicago truncatula*, as a pre-eminent model plant for studying symbiotic relationships between plants and their symbiotic microbes, has been widely used to understand the effect of AM fungi on plant lead stress [19,32]. The split-root system, which separates the AM fungi colonized and non-colonized root segments, is beneficial to compare the direct and long-distance impact of AM fungi on the plant [33,34]. In this study, we used a split-root system (Figure 1a) to separate the AM fungi colonized and non-colonized root segments of *Medicago truncatula* and investigated the influence of AM fungi on root Pb uptake and Pb transfer from roots to shoots. We hypothesized that: (1) AM fungi increase Pb uptake in colonized and non-colonized root segment through the improvement of plant transpiration; (2) AM fungi deliver Pb to colonized root segment; and (3) plant Pb uptake involves the participation of plant aquaporin. To our knowledge, this is the first study using a split-root system to verify the contribution of AM fungi to plant Pb uptake.

## 2. Materials and Methods

### 2.1. Plant Material, Split-Root System, Growth Substrate, and AM Fungal Inoculum

Seeds of *M. truncatula* (Jemalong A17) were kindly provided by Prof. Philipp Franken (Plant Physiology Department, Humboldt University of Berlin). The seeds were soaked in concentrated sulfuric acid for 10 min and washed 5 times using sterile distilled water. Sterilized seeds were germinated in Petri dishes with water agar (0.7%; *w*/*v*) at 4 °C for 4 days, and at room temperature in darkness for 2 days. After placed at room temperature for 1 d in the light, the seeds were germinated [32].

Germinated seeds were transplanted into plastic pots (10 cm in diameter, 12 cm in height) with sterilized sand (<2 mm) to grow roots, and each pot contained one germinated seed. After 8 weeks, uniform seedlings were selected for the pot experiment. The roots were washed with tap water and split in half evenly and then planted in a split-root system consisting of 2 adjoining compartments with one root half in each (as Figure 1a). Each root compartment was filled with 0.8 kg growth substrate. The split-root system was made of acrylic plate bonded with ABS plastic adhesive. The 2 compartments of the system were separated by an acrylic plate to prevent the transfer of Pb and AM fungal inoculum between compartments.

The growth substrate was a mixture of sand and vermiculite (1:1; *v*:*v*), and the contents of available nitrogen, available phosphorus, and available potassium were 10.78 mg/kg, 2.49 mg/kg, and 24.00 mg/kg, respectively. The sand was sieved through a 2 mm sieve, thoroughly washed with tap water, and sterilized at 170 °C for 4 h. The vermiculite was autoclaved at 121 °C (0.11 MPa) for 2 h for sterilization. The vermiculite was a clay mineral with a 2:1 crystalline structure that contained 2 silica tetrahedral sheets with a central alumina octahedral layer [35].

The AM inoculum of *Rhizophagus irregularis* (Bank of Glomales in China, No. BGC BJ09), which consisted of a sandy substrate that contained spores (approximately 21 spores per gram), mycelium, and colonized root fragments, was provided by the Beijing Academy of Agriculture and Forestry Sciences (Beijing, China).

### 2.2. Experimental Design

The experiment consisted of 5 treatments (Figure 1b) that were neither AM inoculum nor Pb application in root compartment (CK), only AM inoculum application in 1 root compartment (OA), only Pb application in 1 root compartment (OP), AM inoculum and Pb applications in separated root compartments (SE), and AM inoculum and Pb applications in the same root compartment together (TO). The seedling roots in different root compartments were also named according to their position, AM status, and Pb status (as in Figure 1b). Ten grams of inoculum was applied underneath the root of *M. truncatula* seedlings upon transplanting into the split-root system in mycorrhizal treatments, whereas sterilized inoculum (170 °C for 4 h) was applied in the non-mycorrhizal treatments. Pb was applied 4 weeks after the AM inoculum application to ensure AM fungal colonization. It was accomplished by applying 32 mL 20 g L^−1^ Pb(NO_3_)_2_ solution to the junction of root and growth substrate by syringe to reach 800 mg kg^−1^ Pb in growth substrate. Four seedlings were merged into 1 sample as 1 biological replication. Each treatment contained 3 biological replications.

Seedlings were grown in a greenhouse at 28 °C/24 °C day/night temperatures under 16 h daylight and 40–60% humidity. Twenty milliliters of modified Hoagland’s nutrient solution [36] containing 10% phosphate (0.1 mM KH_2_PO_4_) was added twice a week to each root compartment before Pb application. After Pb application, only water (20 mL) was added to the root compartment of all treatments once every 2 days to avoid direct precipitation of Pb.

### 2.3. Plant Sampling, Biomass, and AM Fungal Colonization

At harvest (8 weeks after Pb treatment), the biomass of shoots and roots and the fresh-to-dry mass ratio [37] were measured. After measuring fresh weights, parts of the leaves were dried in an oven at 105 °C with forced air circulation for 15 min to inactivate enzymes and then at 65 °C until they reached a constant weight for Pb content measurement. The remaining parts of the leaves were immediately frozen in liquid nitrogen and stored at −80 °C. Roots were soaked with water for root structure scanning (EPSON EXPRESSION 1680, Seiko Epson Corporation, Japan). After root structure scanning, parts of roots were fixed in FAA solution (37% formaldehyde: glacial acetic acid: 95% ethanol, 9:0.5:0.5, *v*:*v*:*v*) for assessment of AM colonization [38]. Total colonization and arbuscular colonization were measured using the magnified cross-section method [39]. Parts of the roots were dried in an oven at 105 °C for 15 min and then at 65 °C with forced air circulation until they reached a constant weight for Pb concentration measurement. The remaining parts of the roots were immediately frozen in liquid nitrogen and stored at −80 °C.

### 2.4. Concentrations of Pb and P

The dry sample was ground in a mortar and placed in the digestion tube (50 mL) with a 5 mL mixture of HNO_3_ plus HClO_4_ (4:1) to digest at a temperature that gradually increased to 220 °C. Lead concentrations were measured using flame atomic absorption spectrometry (PinAAciie 900F, American), and P concentration was measured using the Molybdenum yellow colorimetric method [40]. Lead content was calculated using Pb concentration, fresh-to-dry mass ratio, and plant biomass [37]. The ratio of root Pb concentration to root surface area was calculated to indicate the root surface’s contribution to Pb absorbing capacity.

### 2.5. Photosynthesis

The fifth leaf of each plant was used for the measurements. On the harvest day from 8:00 to 11:30 a.m., the net photosynthetic rate (Pn), intercellular CO_2_ concentration (Ci), transpiration rate (Tr), and conduction to H_2_O (Gs) were measured using a Li-6400 portable open flow gas-exchange system (Li Corporation, American) and converted with measured leaf area. Leaf area was measured by ImageJ 1.38 (National Institutes of Health, American) after being photographed by a camera. The measurement conditions were as follows: photosynthetically active irradiation, 1000 μmol m^−2^ s^−1^; temperature, 22 °C; relative humidity, 30%; and CO_2_ concentration of sample cell, 419 μmol mol^−1^.

### 2.6. Gene Relative Expression

Root samples stored at −80 °C were ground and homogenized with mortar and pestle with liquid nitrogen. Total RNA was isolated from root samples by E.Z.N.A.^TM^ Plant RNA Kit (Omega Biotech, Norcross, GA, USA) following the supplier’s instructions. After quantification of RNA yield by Nanodrop 2000 (Thermo Scientific, Pittsburgh, PA, USA), cDNA was synthesized from 1000 ng of RNA using FastKing RT Kit with gDNase (TIANGEN Biotech, Beijing, China). The synthesized cDNA was diluted 5-fold and used as the template for PCR reactions.

The primers used in the qRT-PCR were as in [26] and are listed in Supplementary Table S1. Gene relative expression was normalized to the *M. truncatula* housekeeping gene *MtEF1α*. The relative expressions of *MtPT4* and *MtBCP1* were used as the indicator of functional arbuscules and their quantity [41,42]. The qRT-PCR reaction was conducted using the CFX96 real-time PCR detection system (Bio-Rad Laboratories, Hercules, CA, USA) and contained 5 μL ChamQ^TM^ Universal SYBR^®^ qPCR Master Mix (Vazyme Biotech, Nanjing, China), 0.5 μL (10 μM) of each primer, 1 μL a cDNA, and 3 μL ddH_2_O. The PCR procedure consisted of a 3 min denaturation at 95 °C; 40 cycles of denaturation at 95 °C for 10 s; annealing at the annealing temperature (Supplementary Table S1) for 20 s; extension at 72 °C for 20 s; followed by heating from 60 to 95 °C to check the specificity of the PCR amplification. All samples were technically replicated twice. Negative controls without cDNA were run within each analysis. The relative quantity of transcripts was determined using the 2^−^^ΔCT^ method [43].

### 2.7. Statistical Analysis

Statistical analysis was performed using the SPSS 19.0 statistical program (SPSS Inc., Chicago, IL, USA). Data were compiled with the assumption of a normal distribution, and the variance equality was also tested. Multiple comparisons were tested by LSD. Correlation analyses were performed using Spearman’s correlation (Supplementary Table S2). Figures were drawn with Origin 2018 (Origin Lab, Northampton, MA, USA). Heatmap and cluster analysis of relative genes were performed using MetaboAnalyst 4.0 [44].

## 3. Results

### 3.1. Biomass and Colonization

**Figure 2:**
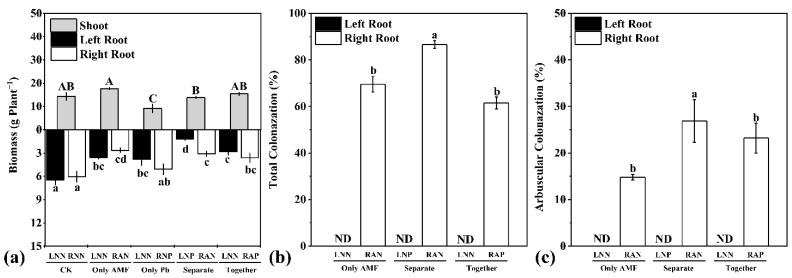
Effects of Pb and *R. irregularis* inoculation on fresh weight of shoots and roots (**a**), total colonization (**b**), and arbuscular colonization (**c**) in *M. truncatula*. The data are shown as means ± standard error (*n* = 3). Different uppercase and lowercase letters above the columns indicate a significant difference among the means by LSD test (*p* < 0.05), respectively. ND = not detected. The abbreviations are consistent with Figure 1.

Eight weeks after Pb application in root compartments, the biomass of shoot and root in different compartments was recorded (Figure 2a). In the CK treatment, the biomass of roots in the two compartments showed no difference, which indicated that the split-root system divided the roots into two parts evenly. Inoculation of *R. irregularis* in one root compartment (comparing OA treatment with CK treatment) increased the shoot biomass (not significantly), but reduced root biomass both locally (OA-RAN) and systemically (OA-LNN). Application of Pb in one root compartment (comparing treatment OP with CK) significantly reduced shoot biomass and root biomass in the other root compartment (OP-LNN). When plants received inoculations of *R. irregularis* and Pb (together and separately), the shoot biomass was not affected significantly (comparing treatment SE and TO with CK), but the root biomass was reduced in both compartments.

No AM fungal feature was observed in roots from the non-mycorrhizal treatment (Figure 2b,c). Over 60% of root in the OA-RAN treatment was colonized, and the typical feature (arbuscules) was observed. Application of Pb showed little limitation to the total colonization of *R. irregularis* in the treatment TO (not significantly), but it significantly promoted the total and arbuscular colonization in the treatment SE. The relative expressions of *MtPT4* and *MtBCP1* that were used as the indicator of functional arbuscules and their quantity [41,42] resembled the colonization results (Supplementary Figure S1).

### 3.2. Root Structure, Pb Concentration, and Content

**Figure 3:**
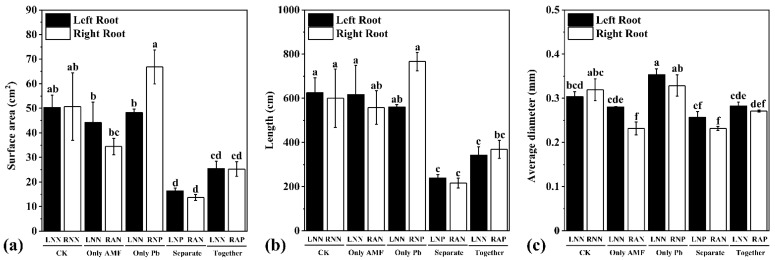
The root surface area (**a**), length (**b**), and average diameter (**c**) of *M. truncatula* in different treatments. The data are the means ± standard error (*n* = 3). Different letters above the columns indicate a significant difference among the means by LSD test (*p* < 0.05), respectively. The abbreviations are consistent with Figure 1.

In the CK treatment, root surface, length, and average diameter in the two compartments showed no difference (Figure 3a–c), which indicated that the spilt-root system divided roots into two parts evenly. Inoculation of *R. irregularis* or Pb application in the OA treatment and OP treatment did not show local or systemic influence on root surface area, length, or average diameter, except for the OA-RAN treatment, in which the average root diameter was locally reduced. The results described above indicated that inoculation of *R. irregularis* directly reduced average root diameter. The root surface area, length, and average diameter were reduced (compared to the CK treatment) when plant roots received *R. irregularis* inoculation and Pb application separately (SE treatment) or together (TO treatment).

**Figure 4:**
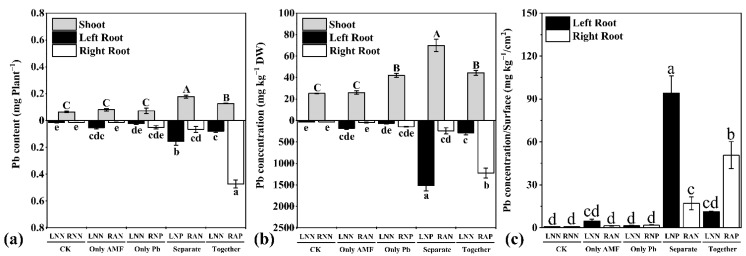
The Pb content (**a**), total Pb concentration (**b**), and the ratio of Pb concentration to surface (**c**) in *M. truncatula* plants in different treatments. The data are the means ± standard error (*n* = 3). Different uppercase and lowercase letters above the columns indicate significant differences among the means by LSD test (*p* < 0.05), respectively. The abbreviations are consistent with Figure 1.

Environmental Pb existed and could not be eliminated as in a previous study [32]. To evaluate the effect of *R. irregularis* on Pb extract and stimulation, both Pb concentration and content were calculated. The lowest concentration and content of Pb in root and shoot were shown in the CK treatment (Figure 4a,b). Solely Pb application increased local root Pb concentration (comparing OP-RNP to CK-RNN by *t*-test, *p* = 0.007) and shoot Pb concentration. The highest concentration and content of Pb in the shoot were shown in the SE treatment. The highest concentration of Pb in the root segment was shown in the SE-LNP root compartment, and the highest content of Pb in the root segment was shown in the TO-RAP root compartment. Inoculation of *R. irregularis* in one root compartment increased Pb concentration in root segments from the other compartment to which extra Pb solution had been added (comparing SE-LNP to OP-RNP) or not (comparing OA-LNN and TO-LNN to CK-RNN; OA-LNN was compared to CK-LNN by *t*-test, *p* = 0.027). The Pb concentrations and contents in root segments from compartments that received Pb were increased compared to CK. Especially, the increases in Pb concentration and content in the root segment were higher when inoculation of *R. irregularis* was involved (comparing SE-LNP and TO-RAP to OP-RNP). In addition, the Pb content in the root segment that had direct contact with *R. irregularis* (TO-RAP) was much higher than that in the root segment that had indirect contact with *R. irregularis* (SE-LNP).

The ratio of root Pb concentration to root surface area was calculated to evaluate the contribution of the plant root surface to Pb uptake (Figure 4c). Compared with CK, increased ratios were observed in the root compartment that received extra Pb (SE-LNP and TO-RAP) and in the root compartment that was inoculated with *R. irregularis* (SE-RAN) in the SE treatment. The highest ratio was shown in the root compartment (SE-RAN) that received Pb and *R. irregularis* in different root compartments.

### 3.3. Photosynthesis

**Figure 5:**
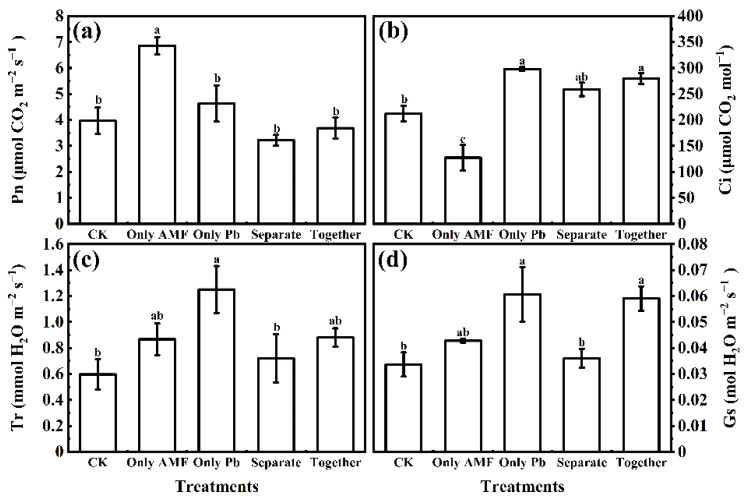
Effects of Pb and *R. irregularis* inoculation on net photosynthetic rate Pn (**a**), intercellular CO_2_ concentration Ci (**b**), transpiration rate Tr (**c**), and stomatal conductance Gs (**d**) in leaves of *M. truncatula*. The data are the means ± standard error (*n* = 3). The different letters above the columns indicate significant differences among the means by LSD test (*p* < 0.05), respectively. The abbreviations are consistent with Figure 1.

Compared to the CK, solely *R. irregularis* inoculation increased Pn but decreased Ci (Figure 5a,b). Lead application (only Pb) increased Tr, while only Pb applied and Pb applied with *R. irregularis* together increased Ci and Gs (Figure 5b–d). When Pb and *R. irregularis* inoculum were applied together in the same root compartment, the Gs was higher than when they were applied separately in different root compartments.

### 3.4. Relative Expressions of Aquaporins

**Figure 6:**
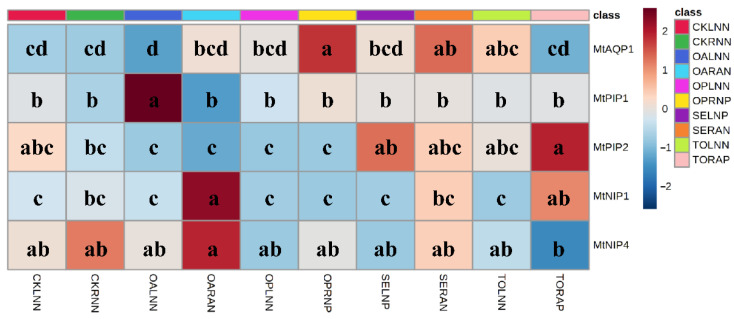
Effects of Pb and *R. irregularis* inoculation on the expression of *MtAQP1*, *MtPIP1*, *MtPIP2*, *MtNIP1,* and *MtNIP4* in root of *M. truncatula*. Expression of *MtEFα* in the root of *M. truncatula* was used for normalization. Different letters within each gene indicate significant differences by LSD test (*p* < 0.05), respectively. The abbreviations are consistent with Figure 1.

To test the hypothesis that inoculation of *R. irregularis* improves the capacity of Pb uptake in *M. truncatula* with the help of aquaporins, the relative expressions of aquaporins were detected (Figure 6). Inoculation of *R. irregularis* locally increased the relative expressions of *MtAQP1* in root compartment SE-RAN, *MtPIP2* in root compartment TO-RAP, and *MtNIP1* in root compartments OA-RAN and TO-RAP (compared to CK). Inoculation of *R. irregularis* also systemically increased the relative expression of *MtPIP1* in root compartment OA-LNN. Application of Pb locally increased the relative expression of *MtAQP1* in root compartment OP-RNP, *MtPIP2* in root compartment TO-RAP, and *MtNIP1* in root compartment TO-RAP (compared to CK). The relative expression of *MtNIP4* was higher in root compartment OA-RAN than that in root compartment TO-RAP.

### 3.5. Correlation Analysis

From the Spearman correlation analysis (Supplementary Table S2), Pb concentration and content in shoot and root showed negative correlations with root biomass, root surface area, and root length, but positive correlations with the relative expression of *MtPIP2* in the root. Moreover, root Pb concentration showed a positive correlation with the relative expression of *MtPT4*. The ratio of root Pb concentration to root surface area showed positive correlations with Pb concentration and content of shoot and root. Additionally, the ratio of root Pb concentration to root surface area showed positive correlations with the relative expression of *MtPT4*, *MtBCP1*, and *MtPIP2* in the root.

## 4. Discussion

Arbuscular mycorrhizal fungi can survive in various environments including Pb polluted areas and improve growth and stress tolerance of host plants [15,32,45]. Under Pb stress, AM fungi colonized plants were reported to have better growth [17,18,46] and accumulate more Pb in root than in shoot [31]. To verify the influence of AM fungi on plant root Pb uptake, a split-root system was established to separate the colonized and non-colonized root segments and compare their differences in Pb uptake.

The evenly distributed root biomass in two root compartments of CK treatment demonstrated the success of the split-root system. A similar split-root system was used in other studies of the systemic influence of AM fungi in *M. truncatula* [33,34]. When root segments of *M. truncatula* were only colonized in one root compartment, the AM fungi showed improvement on plant shoot growth and systemic reduction of plant root growth (Figure 2a) as reported previously [33]. The systemic influence of AM fungi on root growth reduction was due to the carbon investment of plants in AM fungal hyphae, which require less carbon than root to acquire nutrients and water from soil [47]. Plant carbon expenditure on AM fungi was more economic than expenditure on root growth in terms of improving P uptake [48]. Application of Pb had a negative effect on the biomass accumulation of non-AM plants, confirming the sensitivity of *M. truncatula* to Pb toxicity in the treated concentration [19]. The beneficial effect and alleviation of Pb toxicity by AM fungi, which was indicated by the higher shoot biomass of AM plants than those of the non-AM plants, was consistent with previous studies [18,30,32].

Inoculation with *R. irregularis* successfully established AM symbiosis in the root compartments and set the basis for this study. The colonization rate showed a similar tendency with the relative expression of *MtPT4* and *MtBCP1* in the root segment, and this supported the view that the expressions of these two genes were indicators of AM symbiosis in the root of *M. truncatula* [34,41,42]. When Pb and *R. irregularis* were applied in different root compartments, the colonization rate of *R. irregularis* increased [49] and the relative expressions of *MtPT4* and *MtBCP1* were up-regulated. This might be because AM symbiosis maintains the balance of plant mineral element uptake, which was disturbed by Pb through hindering permeability of root cell plasma membrane [50,51], by increased reliance of plant on AM symbiosis [27,52]. Nevertheless, when Pb and *R. irregularis* were applied in the same root compartment, the extension of extraradical hyphae was inhibited. Moreover, the reliance of plants on AM symbiosis was limited, and the colonization rate was restored. Heavy metal presence in the substrate can reduce AM fungal hyphae extension during spore germination in vitro [53]. This could have happened when Pb and *R. irregularis* were applied in the same root compartment. The extension of extraradical hyphae may have been inhibited by Pb with the reliance of the plant on AM symbiosis being limited, and the colonization rate being restored to the OA-RAN compartment value.

Although Pb is a non-essential element, the uptake of Pb by a plant is inevitable [5,6,54]. The application of Pb in one root compartment hardly affected the root Pb concentration in the other root compartment, but increased the shoot Pb concentration as [55] shown with Cd. Compared with non-mycorrhizal plants, mycorrhizal plants usually had higher shoot Pb content under Pb stress. This is related to *R. irregularis* which improves plant growth and reduces Pb toxicity. When *R. irregularis* and Pb were applied in different root compartments, the Pb concentration and content in the shoot were also increased, and this indicated an improvement of Pb transfer from non-colonized root segment to shoot by AM symbiosis [17]. However, the Pb concentration and content in the shoot of TO treatment was lower than those of SE treatment (Figure 4a,b), implying an inhibitory effect of *R. irregularis* on Pb translocation.

In the root, Pb application increased the Pb concentration and content (Figure 4a,b). The increase in Pb concentration and content in colonized and non-colonized *M. truncatula* root segments by *R. irregularis* (comparing TO-RAP and SE-LNP to OP-RNP) (Figure 4a,b) followed a previous study showing that AM plants accumulated more Pb in root than non-mycorrhizal plants [30]. The increased root Pb concentration and content by *R. irregularis* might be due to the increased water and nutrient uptake, which was proven by the higher shoot biomass and lower root biomass of SE and TO treatments compared to those of OP treatment (Figure 2a) and by the positive correlations of the relative expression of *MtPT4* and root Pb content. Moreover, the increase in root Pb content by *R. irregularis* in colonized root segment was higher than that in the non-colonized root segment (comparing TO-RAP with SE-LNP) (Figure 4a). It might be due to the mycorrhizal diluting effect [56]. This indicated an increased Pb accumulation capability of AM fungi colonized root segment, which has two nutrient and water uptake pathways [27,28].

**Figure 7:**
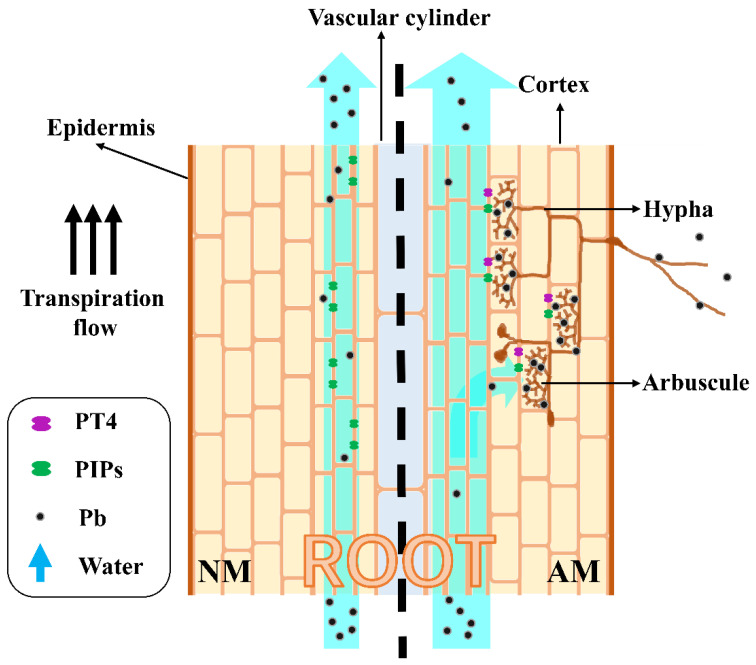
AM fungi can immobilize Pb that *R. irregularis* in the inoculated compartment as a sifter reduced the content of Pb in a plant.

The Pb accumulation capability of different root segments was compared through the ratio of root Pb concentration to root surface area. The ratio was positively correlated with the relative expressions of *MtPT4* and *MtBCP1*. This result further confirmed that the AM fungi increased the Pb accumulation capability of plant roots. However, the Pb concentration and content in the shoot of TO treatment was lower than those of SE treatment (Figure 4a,b) and the ratio of root Pb concentration to root surface area in TO-RAP treatment was lower than that in SE-LNP treatment (Figure 4c), implying an inhibitory effect of AM fungi on Pb translocation. It may due to the increase in P by AM fungi (Supplementary Figure S2). Pb(PO_4_)_2_ is a stable environmental soil Pb form that may form rapidly when adequate phosphate is present [57]. The retention of Pb by AM fungi might also be the result of sequestration by their cell walls and proteins and compartmentation in vacuoles [27,58]. The AM fungi colonized root segments with the mycorrhizal pathway transferred less Pb to shoot than non-colonized root segments without the mycorrhizal pathway. Thus, the Pb supplied by the colonized root segment with the mycorrhizal pathway to plant root has lower mobility than Pb absorbed from growth substrate by plant root itself (Figure 7).

The nutrient delivery of the mycorrhizal pathway was ascertained to follow the water flow [29,59], which involves the participation of aquaporins [25]. The increased effect of AM fungi on Pb and P showed a similar phenomenon. (Supplementary Figure S2). The AM fungi inoculation both systemically and locally increased root P uptake. The increased P uptake from AM fungi inoculation was inhibited by Pb, especially in SE-LNP (not significant). This also indicated that AM plant might have promoted Pb uptake as P uptake by providing more transpiration flow.

The relative expression of gene encoding MtPIP2, which was suggested to have higher water permeability than PIP1 and form a heterotetramer with PIP1 [60], was up-regulated in root compartment TO-RAP (Figure 6) and was positively correlated with the Pb content and concentration in root and shoot and with the ratio of root concentration to root surface area. This result fitted the hypothesis that Pb uptake by plant root follows water flow (Figure 7). The specific role of MtPIP2 in Pb uptake is under study.

## 5. Conclusions

To summarize, inoculation with *R. irregularis* had a beneficial effect on *M. truncatula* and could alleviate Pb toxicity. AM symbiosis increased Pb uptake in both colonized and non-colonized plant root segments, whereas AM fungi might transfer extra Pb to the colonized root segment. The Pb uptake through the colonized root segment had low mobility moving from root to shoot, and might be sequestrated and compartmented by AM fungi. The Pb uptake of plant roots might follow water flow, which is facilitated by the aquaporin MtPIP2. Further research will quantify the Pb that is directly transferred from *R. irregularis* to plant root, and decipher the role of MtPIP2 in root Pb uptake.

## Data Availability

Not applicable.

## Supplementary Materials

The following are available online at https://www.mdpi.com/article/10.3390/microorganisms9061203/s1, Figure S1: Effects of Pb and *R. irregularis* inoculation on the expressions of *MtPT4* (**a**) and *MtBCP1* (**b**) in the root of *M. truncatula*, Figure S2: Effects of Pb and *R. irregularis* inoculation on the concentration of P in roots and shoots of *M. truncatula*. Table S1: Primer list of RT-qPCR. Table S2: Correlation analysis between Pb and AM fungi colonization in root with biomass, root structure, nutritive element concentrations, and the relative expressions of genes in *M. truncatula* plants in different treatments.
