# Supplementary material for: Arbuscular Mycorrhizal Fungi Increase Pb Uptake of Colonized and Non-Colonized Medicago truncatula Root and Deliver Extra Pb to Colonized Root Segment
Source: Microorganisms. 2021 Jun 2;9(6):1203. doi: 10.3390/microorganisms9061203 (PMC8229133; doi:10.3390/microorganisms9061203)
Supplement: Supplementary file 1 [file microorganisms-09-01203-s001.zip › Supplementary caption.pdf]

Supplementary Materials:

Figure S1 Effects of Pb and *R. irregularis* inoculation on the expressions of *MtPT4* **(a)** and *MtBCP1* **(b)** in the root of *M. truncatula*. Expression of the *MtEFα* in the root of *M. truncatula* was used as an internal control for normalization. The data are the means ± standard error (n = 3). Different letters within each gene indicate significant differences by LSD test ($P < 0.05$), respectively. The abbreviation is consistent with Fig.2.

Figure S2 Effects of Pb and *R. irregularis* inoculation on the concentration of P **(a)**, K **(b)**, Ca **(c),** and Mg **(d)** in roots and shoots of *M. truncatula*. The data are the means ± standard error (n = 3). Different letters within each gene indicate significant differences by LSD test ($P < 0.05$), respectively. The abbreviation is consistent with Fig.2.

Table S1 Primer list of RT-qPCR.

Table S2 Correlation analysis between Pb and AM fungi colonization in root with biomass, root structure, nutritive element concentration, and the relative expressions of genes in *M. truncatula* plants in different treatments.
