# Supplementary figures and images for: Arbuscular Mycorrhizal Fungi Increase Pb Uptake of Colonized and Non-Colonized Medicago truncatula Root and Deliver Extra Pb to Colonized Root Segment
Source: Microorganisms. 2021 Jun 2;9(6):1203. doi: 10.3390/microorganisms9061203 (PMC8229133; doi:10.3390/microorganisms9061203)

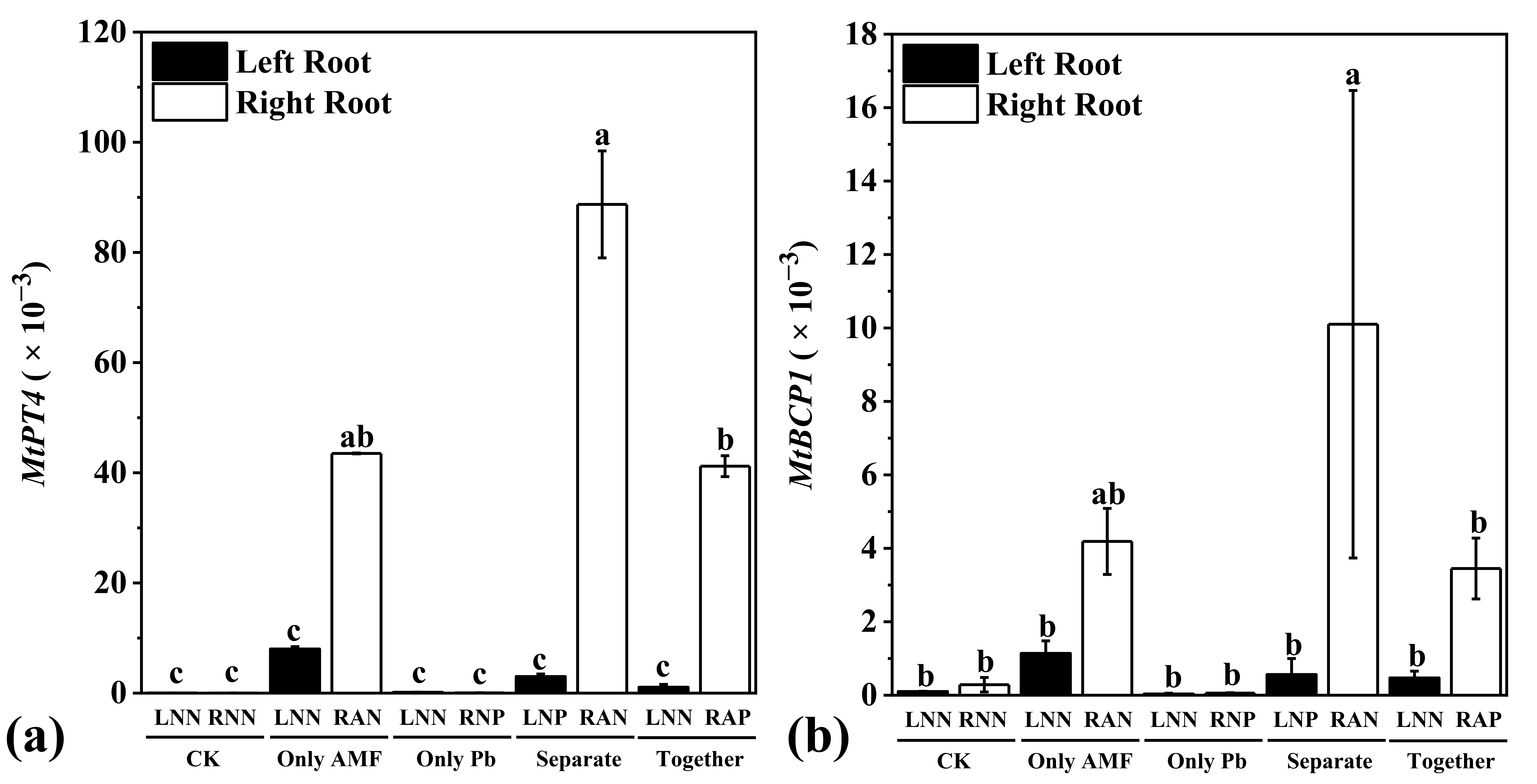

Supplement: Supplementary file 1 [file microorganisms-09-01203-s001.zip › FigureS1.tif]

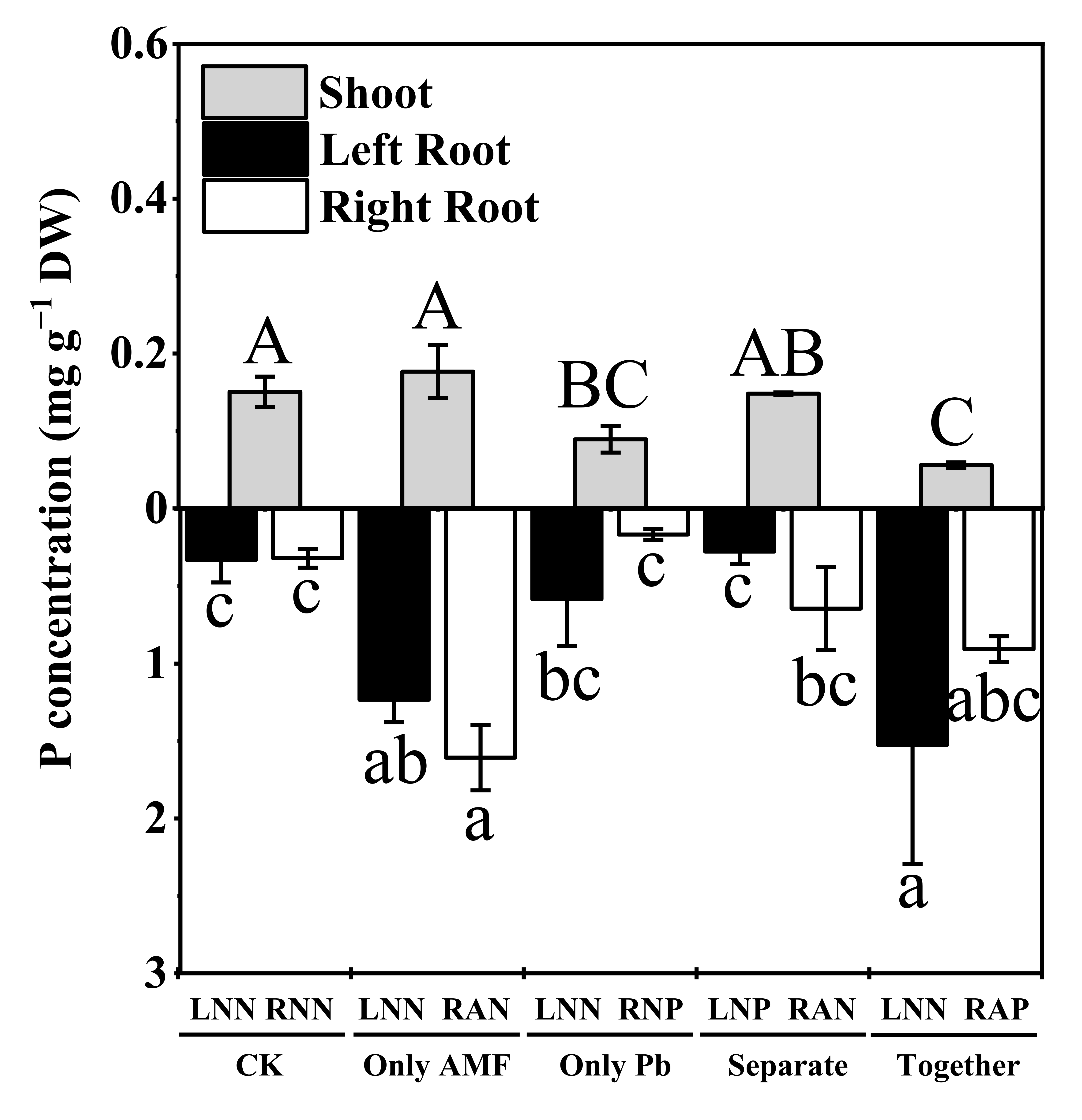

Supplement: Supplementary file 1 [file microorganisms-09-01203-s001.zip › FigureS2.tif]
